# 

**Published:** 1861

**Authors:** 


### Contents---April & May, 1861.

### ORIGINAL CONTRIBUTIONS.

PAGE

ART. I. Remarks on the General Treatment of Strangulated Hernia, with a report of two cases. By A. FISHER, M. D., Chicago........ 193

ART. II. Case, Illustrating evil effects of Veratrum Viride. By E. S. McINTIRE, M. D., Dallas City, Ills........ 200

ART. III. Clinical Lecture on Chronic Gastritis, Post-Mortem Appearances, Inflammation of the Kidneys, etc. By N. S. DAVIS, M. D........ 202

ART. IV. Lectures on Military Surgery. By E. ANDREWS, A. M. M. D., Professor of Surgery, etc........ 249

### SELECTIONS.

Inversion of the Womb. R. C. HAMILL, M. D........ 207

Discussion on Inversion of the Uterus, in the New York State Medical Society........ 212

Some observations on the treatment of the Narrow and Irritable Stricture of the Urethra, D. D. SLADE, M. D........ 221

### BOOK NOTICES.

Transactions of the Ohio State Medical Society........ 226

DALTON'S Human Physiology. Second Edition........ 227

A Treatise on Fevers. ROBERT D. LYONS, K. C. C., etc., etc........ 229

Report on Morbus Coxarius. LEWIS A. SAYRE, M. D., etc........ 229

Summary of Medical Science. Edited by WALTER S. WELLS, M. D........ 230

A Paper on Diphtheria. JAMES WYNNE, M. D........ 231

### EDITORIAL.

An Explanation. Prospects for the Future........ 231

Surgeons and Assistant Surgeons in the Army........ 232

Report of Board of Medical Examiners to the Legislature........ 233

Appointment. Obituary........ 236

American Medical Association. Bellevue Hospital College.,........ 237

Homœopathy in the Michigan University. Letter from Professor PALMER........ 238

Dewitt County Medical Society,........ 239

Personal; Professor MEIGS, Dr. KEATING, Dr. JENNER, Dr. HARRIS........ 240

Naval Medical Examiners. Item........ 241

A Suggestion........ 242

### EDITORIAL ABSTRACTS AND SELECTIONS.

Louisiana Legislature on Quacks. Nitric Acid in Intermittent Fever........ 243

Steadine........ 244

Turpentine as an Anæsthetic. Malt for Children. Chlorate of Potash in Phthisis........ 245

Maxims of Military Surgery........ 246

